# Supplementary material for: Direct evidence for transport of RNA from the mouse brain to the germline and offspring
Source: BMC Biol. 2020 Apr 30;18:45. doi: 10.1186/s12915-020-00780-w (PMC7191717; doi:10.1186/s12915-020-00780-w)
Supplement: Supplementary file 1 — Additional file 1: Figure S1.pENN.AAV.CB7.CI.MIR941.rBG. Plasmid produced for viral vector production by Penn Vector Core. [file 12915_2020_780_MOESM1_ESM.docx]

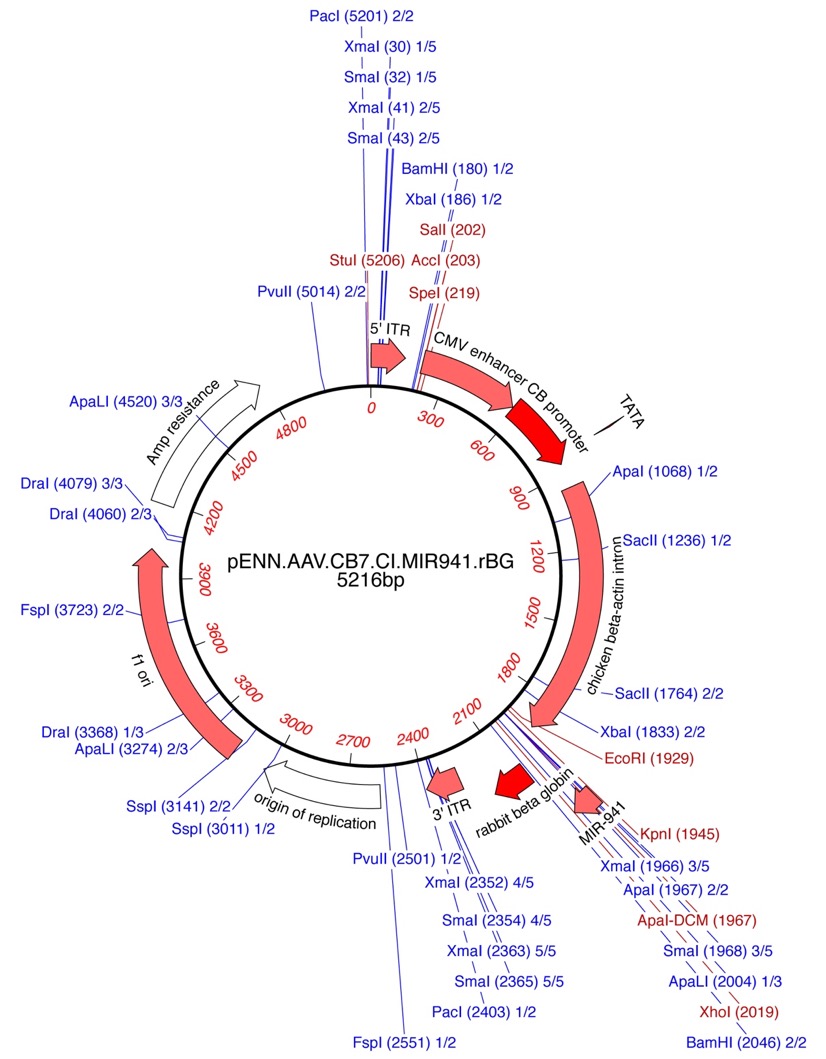


**Additional File 1: Fig. S1.** pENN.AAV.CB7.CI.MIR941.rBG. Plasmid produced for viral vector production by Penn Vector Core.
