# Supplementary material for: Direct evidence for transport of RNA from the mouse brain to the germline and offspring
Source: BMC Biol. 2020 Apr 30;18:45. doi: 10.1186/s12915-020-00780-w (PMC7191717; doi:10.1186/s12915-020-00780-w)
Supplement: Supplementary file 2 — Additional file 2: Figure S2. Detection of (A) MIR941 and (B) a rabbit β-globin fragment in treated adult male mice. Positive control striatal injection sites from adult male mice were used to detect single bands using a gene-specific locked nucleic acid (LNA) quantitative PCR (qPCR) system. [file 12915_2020_780_MOESM2_ESM.docx]

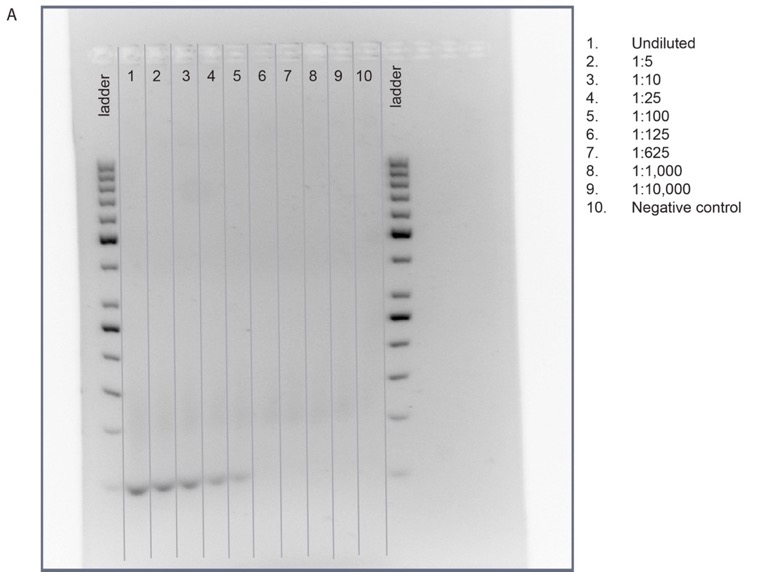


**
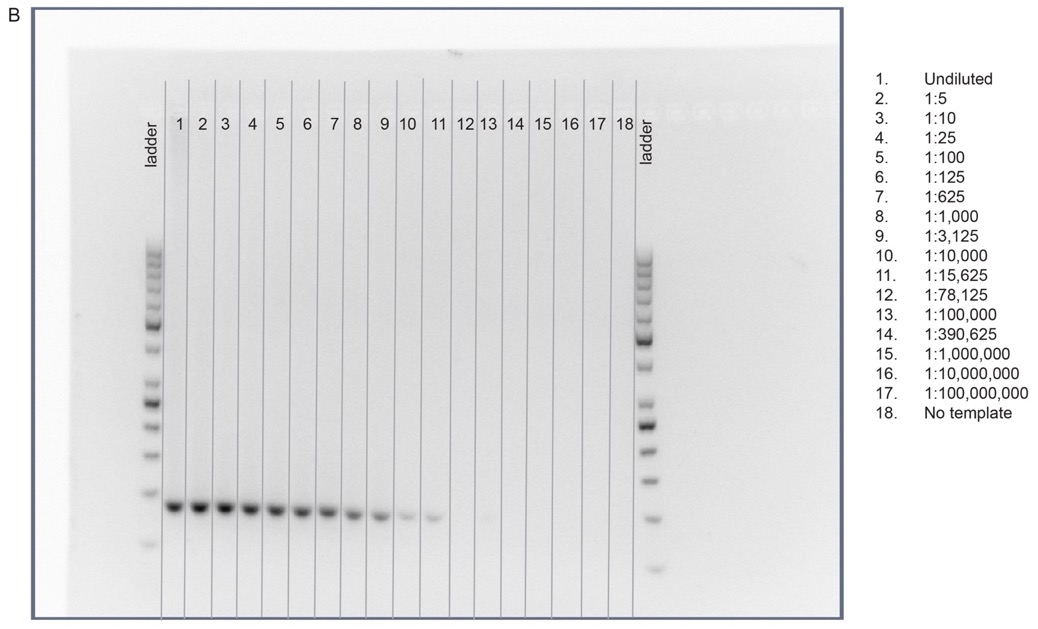
**

**Additional File 2: Fig. S2.** Detection of (**A**) MIR941 and (**B**) a rabbit β-globin fragment in treated adult male mice. Positive control striatal injection sites from adult male mice were used to detect single bands using a gene-specific locked nucleic acid (LNA) quantitative PCR (qPCR) system.
