# Supplementary material for: Direct evidence for transport of RNA from the mouse brain to the germline and offspring
Source: BMC Biol. 2020 Apr 30;18:45. doi: 10.1186/s12915-020-00780-w (PMC7191717; doi:10.1186/s12915-020-00780-w)
Supplement: Supplementary file 3 — Additional file 3: Figure S3. No detection of MIR941 or rabbit β-globin fragment in control (uninjected) or mock-treated (saline-injected) animals at either (A) 8 weeks or (B) 16 weeks post-injection. Additionally, no presence of rabbit β-globin fragment was found in control embryos, collected from 3 separate litters, from uninjected parents (C); positive control 1- MDA cells, positive control 2 – HEK cells; negative control – no template control). [file 12915_2020_780_MOESM3_ESM.docx]

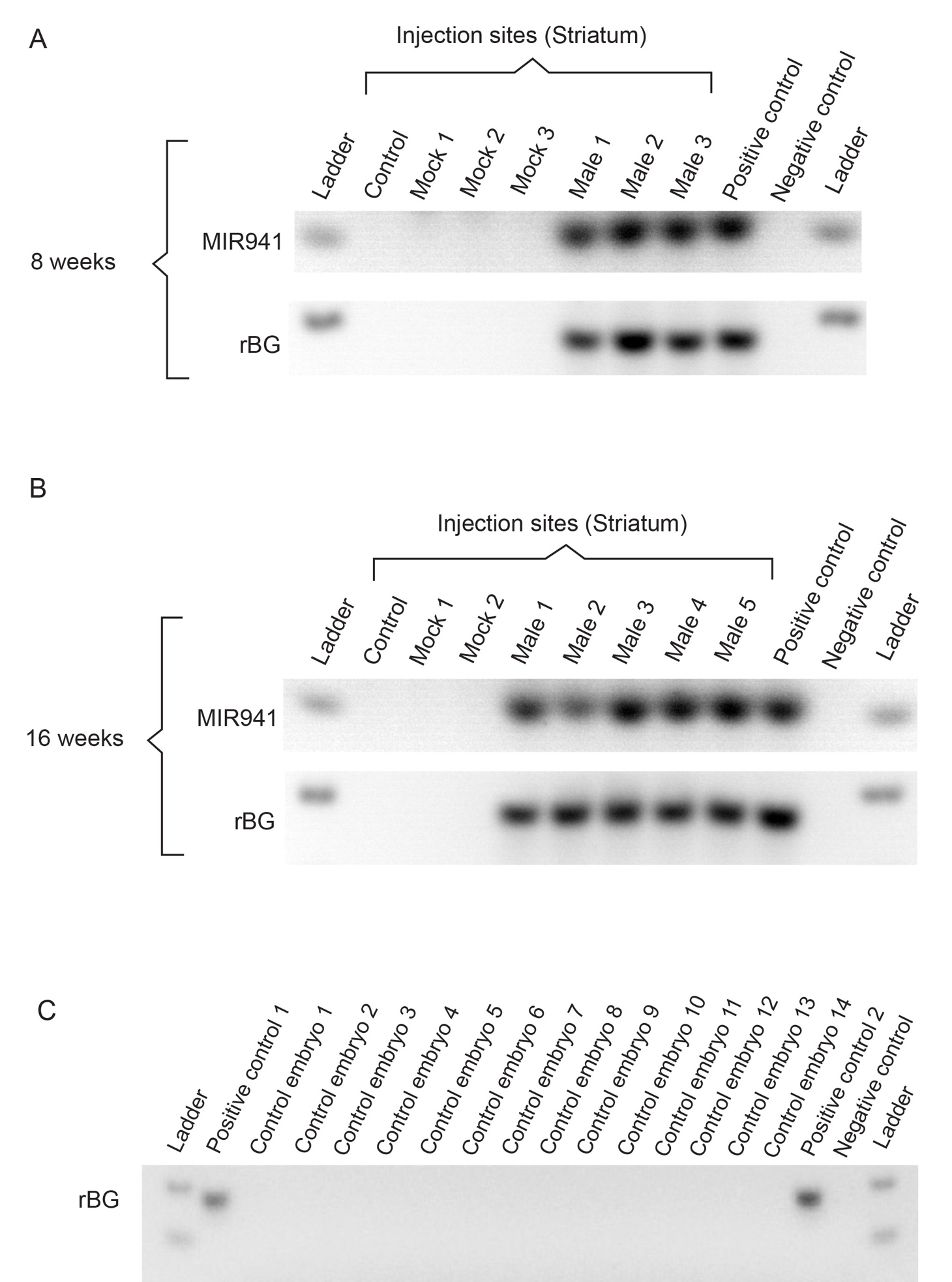


Additional File 3: Fig. S3. No detection of MIR941 or rabbit β-globin fragment in control (uninjected) or mock-treated (saline-injected) animals at either (A) 8 weeks or (B) 16 weeks post-injection. Additionally, no presence of rabbit β-globin fragment was found in control embryos, collected from 3 separate litters, from uninjected parents (C); positive control 1- MDA cells, positive control 2 – HEK cells; negative control – no template control).
