# Supplementary figures and images for: Direct evidence for transport of RNA from the mouse brain to the germline and offspring
Source: BMC Biol. 2020 Apr 30;18:45. doi: 10.1186/s12915-020-00780-w (PMC7191717; doi:10.1186/s12915-020-00780-w)

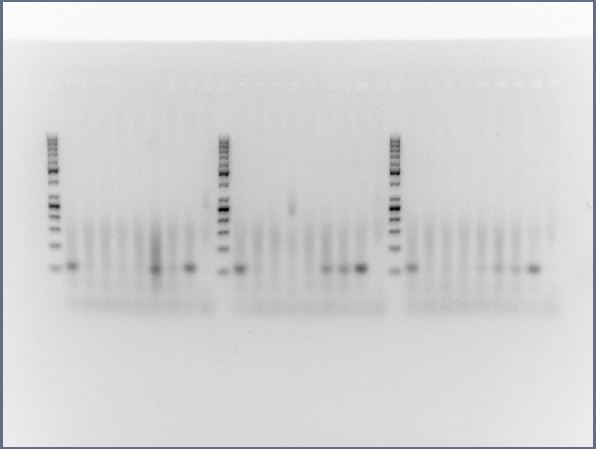


Additional File 4: Fig. S4. Full size, unedited gels used for Figure 1 in the main text.

Supplement: Supplementary file 4 — Additional file 4: Figure S4. Full size, unedited gels used for Fig. 1 in the main text. [file 12915_2020_780_MOESM4_ESM.docx]
