# Supplementary material for: Direct evidence for transport of RNA from the mouse brain to the germline and offspring
Source: BMC Biol. 2020 Apr 30;18:45. doi: 10.1186/s12915-020-00780-w (PMC7191717; doi:10.1186/s12915-020-00780-w)
Supplement: Supplementary file 5 — Additional file 5. Supplementary Information. [file 12915_2020_780_MOESM5_ESM.docx]

**Additional File 5- Supplementary Information**

**Melting temperatures (T_m_) for (*) positive calls for MIR941 on the gels (Figures 1-3).**

The following represent positive calls made in the main text as an asterix (*) in figures 1-3. For a ‘positive’ call, there needed to be a correct size band on the gel (products generated by LNA qPCR), a graphical ‘positive’ via melt curve analysis and T_m_ values that were consistent with positive control values in each run.

*(All other T_m_ values were either significantly divergent from these ‘positive’ values or ‘undetermined’)*

**Figure 1: 2 weeks**

T_m_ (Injection sites): (Male 1) 72.953, (Male 2) 72.834, (Male 3) 72.834

T_m_ (Lymph nodes): (Male 1) 72.114, (Male 2) 72.114, (Male 3) 72.354

T_m_ (Vas/Epididymis): (Male 1) 72.354, (Male 2) 72.474, (Male 3) 71.994

T_m_ (Positive controls): (Male 1) 73.073, (Male 2) 73.073, (Male 3) 72.953

**Figure 2: 8 weeks**

T_m_ (Injection sites): (Male 1) 72.867, (Male 2) 72.986, (Male 3) 72.629

T_m_ (Contralateral site): (Male 2) 72.509

T_m_ (Cerebellum): (Male 1) 72.271, (Male 2) 73.462

T_m_ (Lymph nodes): (Male 1) 72.867, (Male 2) 72.033, (Male 3) 71.914

T_m_ (Vas/Epididymis): (Male 1) 72.986, (Male 2) 71.795, (Male 3) 72.509

T_m_ (Positive controls): (Male 1) 73.224, (Male 2) 73.105, (Male 3) 73.105

T_m_ (Embryos):

- Male 1 - (Embryo 2) 73.224, (Embryo 5) 73.224, (Embryo 6) 72.748, (Embryo 8) 72.629
- Male 2 - (Embryo 1) 72.629
- Male 3 - (Embryo 1) 72.309, (Embryo 2) 72.309, (Embryo 3) 73.343, (Embryo 4) 72.748

**Figure 3: 16 weeks**

T_m_ (Injection sites): (Male 1) 71.636, (Male 2) 71.516, (Male 3) 71.796, (Male 4) 71.396, (Male 5) 71.396.

T_m_ (Contralateral site): (Male 1) 71.996, (Male 2) 72.236, (Male 4) 71.516

T_m_ (Cerebellum): (Male 1) 70.197

T_m_ (Lymph nodes): (Male 1) 71.156, (Male 2) 70.916, (Male 4) 73.548, (Male 5) 71.156

T_m_ (Vas/Epididymis): (Male 3) 73.548, (Male 4) 71.156, (Male 5) 71.156

T_m_ (Positive controls): (Male 1) 71.636, (Male 2) 71.636, (Male 3) 71.796, (Male 4) 71.636, (Male 5) 71.636

T_m_ (Embryos):

- Male 1 - (L2 Embryo 1) 71.156, (L2 Embryo 5) 71.636, (L2 Embryo 8) 71.756
- Male 2 - (Embryo 1) 71.036, (Embryo 5) 71.156
- Male 3 - (Embryo 2) 71.445, (Embryo 5) 72.964, (Embryo 7) 72.146
- Male 4 - (Embryo 1) 71.996, (Embryo 3) 72.356, (Embryo 5) 71.276
- Male 5 - (L1 Embryo 2) 71.516, (L1 Embryo 3) 70.916, (L1 Embryo 7) 70.916, (L2 Embryo 3) 71.036, (L2 Embryo 6) 71.756, (L2 Embryo 7) 71.036
