# Supplementary material for: Direct evidence for transport of RNA from the mouse brain to the germline and offspring
Source: BMC Biol. 2020 Apr 30;18:45. doi: 10.1186/s12915-020-00780-w (PMC7191717; doi:10.1186/s12915-020-00780-w)
Supplement: Supplementary file 6 — Additional file 6: Figure S5. Representative full panel of melt curves for Week 2, Male 1 showing MIR941 LNA qPCR. Positive (black writing) and negative (red writing) melt curves and associated melting temperatures (Tm) are shown and correspond to the bands in Fig. 1. We have used an asterix (*) to denote a positive band of correct size on the gel that also had positive melt curves and Tm values as shown in this figure. [file 12915_2020_780_MOESM6_ESM.docx]

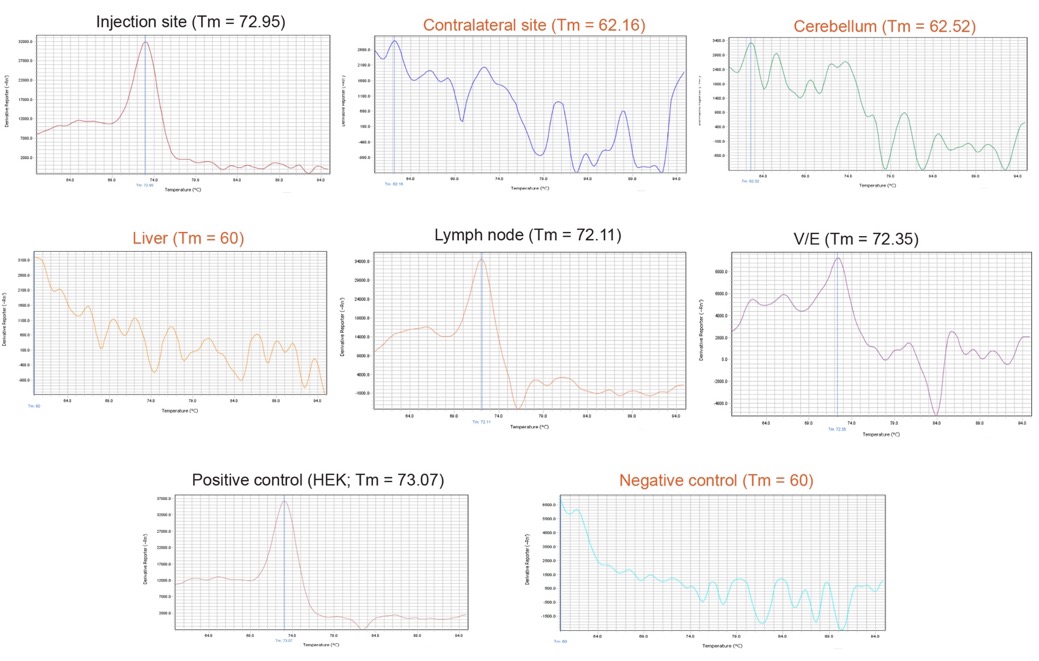


Additional File 6: Fig. S5. Representative full panel of melt curves for Week 2, Male 1 showing MIR941 LNA qPCR. Positive (black writing) and negative (red writing) melt curves and associated melting temperatures (T_m_) are shown and correspond to the bands in Figure 1. We have used an asterix (*) to denote a positive band of correct size on the gel that also had positive melt curves and T_m_ values as shown in this figure.
