# Supplementary material for: Direct evidence for transport of RNA from the mouse brain to the germline and offspring
Source: BMC Biol. 2020 Apr 30;18:45. doi: 10.1186/s12915-020-00780-w (PMC7191717; doi:10.1186/s12915-020-00780-w)
Supplement: Supplementary file 7 — Additional file 7: Figure S6. Full size, unedited gels used for Fig. 2 in the main text. (A) MIR941-Male 1, (B) Rabbit β-globin fragment - Male 1, (C) MIR941-Male 2, (D) Rabbit β-globin fragment - Male 2, (E) MIR941-Male 3, (F) Rabbit β-globin fragment - Male 3. [file 12915_2020_780_MOESM7_ESM.docx]

A


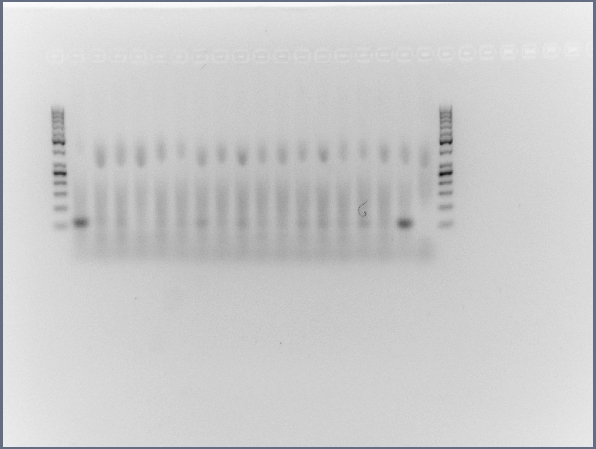


B


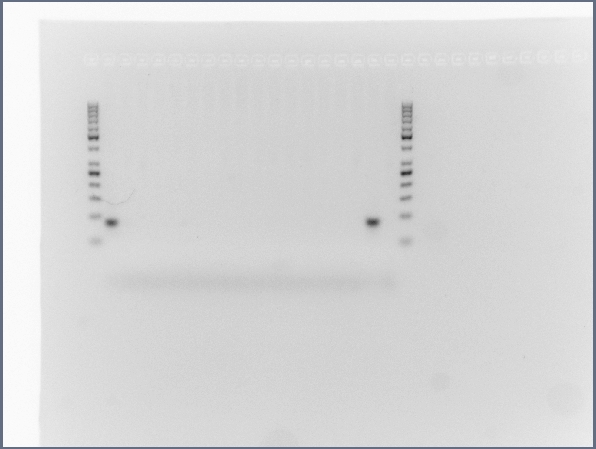


C


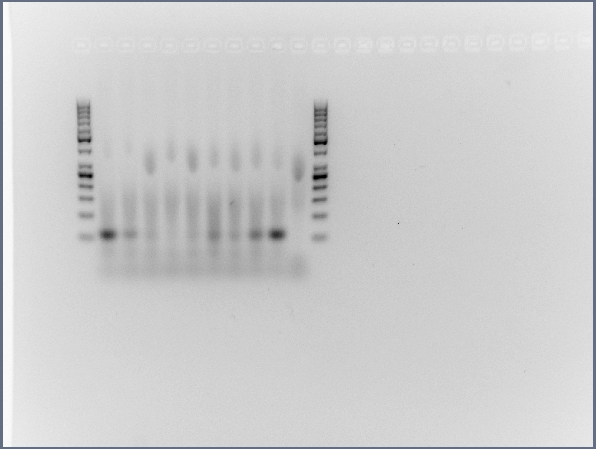
]

D


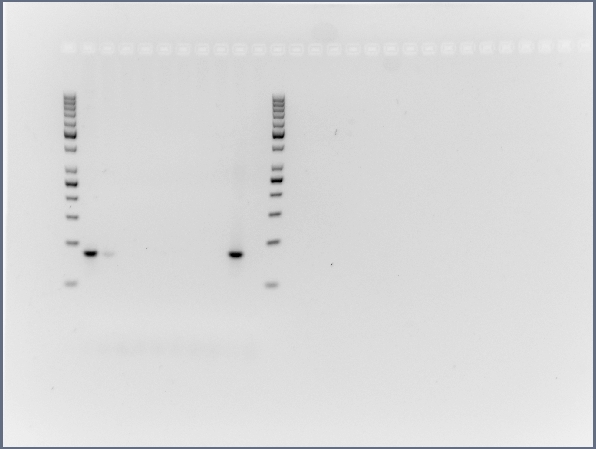


E


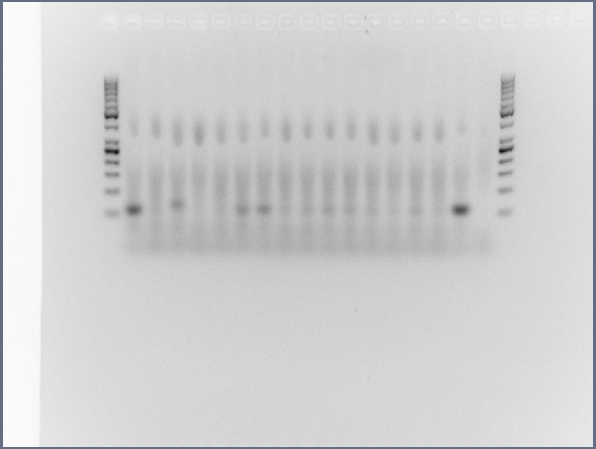


F


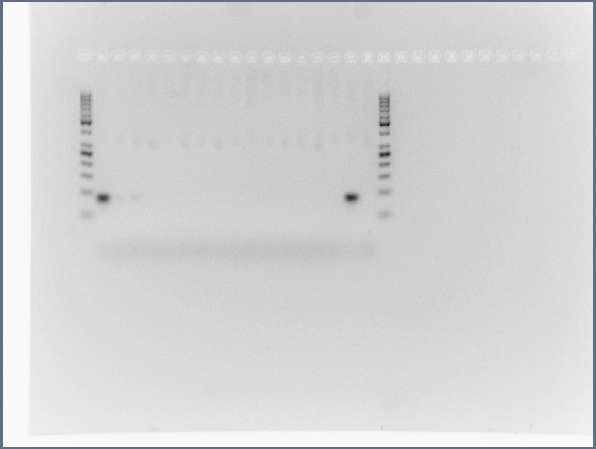


Additional File 7: Fig. S6. Full size, unedited gels used for Figure 2 in the main text. (A) MIR941-Male 1, (B) Rabbit β-globin fragment - Male 1, (C) MIR941-Male 2, (D) Rabbit β-globin fragment - Male 2, (E) MIR941-Male 3, (F) Rabbit β-globin fragment - Male 3.
