# Supplementary material for: Direct evidence for transport of RNA from the mouse brain to the germline and offspring
Source: BMC Biol. 2020 Apr 30;18:45. doi: 10.1186/s12915-020-00780-w (PMC7191717; doi:10.1186/s12915-020-00780-w)
Supplement: Supplementary file 10 — Additional file 10: Figure S9. Cropped (A) figures showing LNA qPCR products for MIR941 (top panel) and rabbit β-globin fragment (bottom panel) from a series of microcentrifuge and ultracentrifuge spins to isolate various fractions of whole blood in five 16 week treated male mice and one age-matched control and mock-treated male. Spins were (1) 3000 x g, (2) 12,000 x g and (3) 110,000 x g. Full size, unedited gels are shown in (B) MIR941 and (C) rabbit β-globin fragment. [file 12915_2020_780_MOESM10_ESM.docx]

A


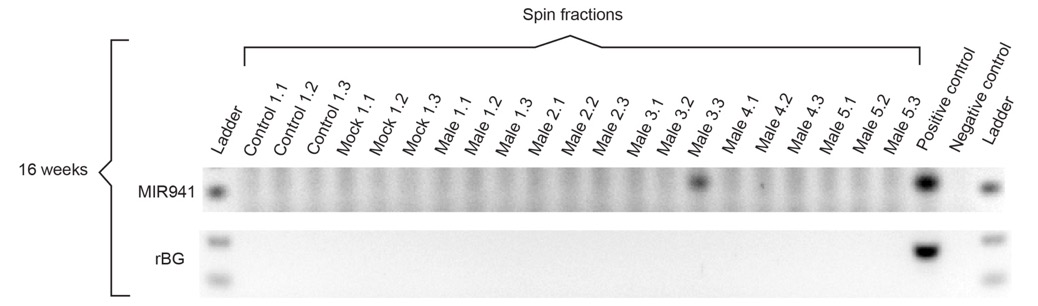


B


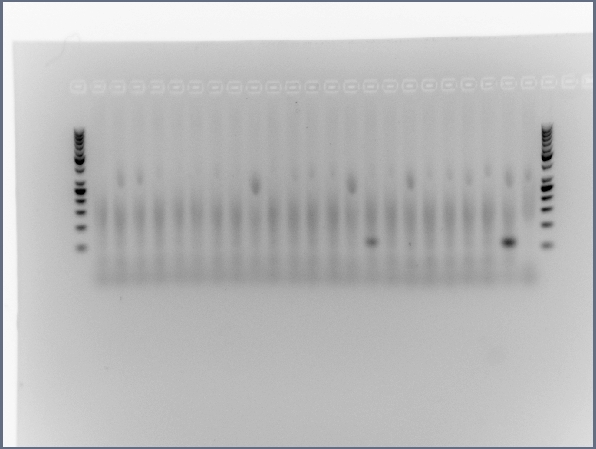


C


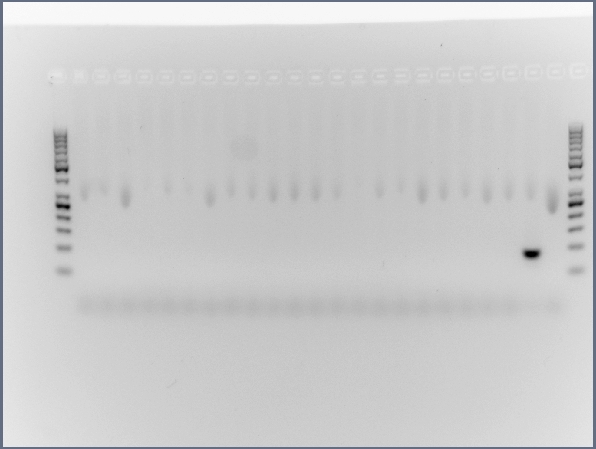


Additional File 10: Fig. S9. Cropped (A) figures showing LNA qPCR products for MIR941 (top panel) and rabbit β-globin fragment (bottom panel) from a series of microcentrifuge and ultracentrifuge spins to isolate various fractions of whole blood in five 16 week treated male mice and one age-matched control and mock-treated male. Spins were (1) 3,000 x g, (2) 12,000 x g and (3) 110,000 x g. Full size, unedited gels are shown in (B) MIR941 and (C) rabbit β-globin fragment.
