# Supplementary material for: Direct evidence for transport of RNA from the mouse brain to the germline and offspring
Source: BMC Biol. 2020 Apr 30;18:45. doi: 10.1186/s12915-020-00780-w (PMC7191717; doi:10.1186/s12915-020-00780-w)
Supplement: Supplementary file 11 — Additional file 11: Figure S10. Higher overall expression of MIR941 expression in the injected sites of 8 week animals compared to both 2 and 16 weeks. A UniSp6 spike-in control was used to normalize expression. [file 12915_2020_780_MOESM11_ESM.docx]

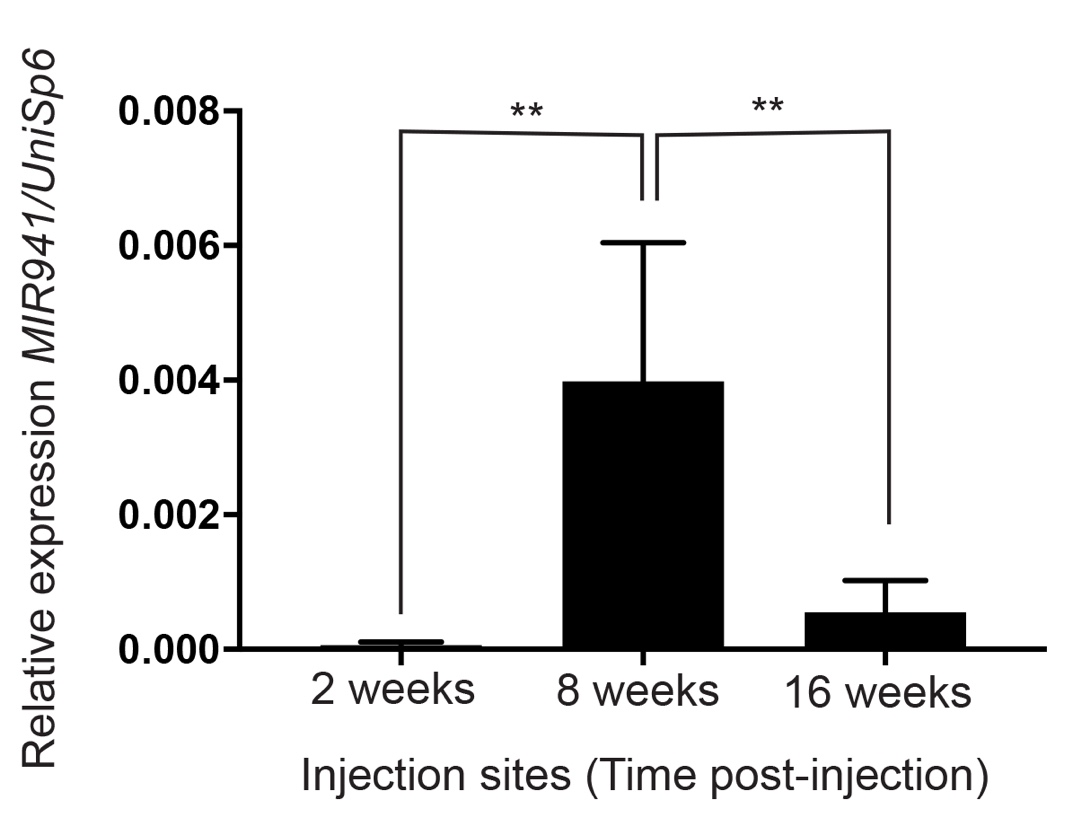


**Additional File 11: Fig. S10.** Higher overall expression of MIR941 expression in the injected sites of 8 week animals compared to both 2 and 16 weeks. A UniSp6 spike-in control was used to normalize expression.
